# Exploring the risk factors for relaparotomy following cesarean delivery

**DOI:** 10.1007/s00404-025-08199-w

**Published:** 2025-10-03

**Authors:** Liri Lewi, Gil Gutvirtz, Tamar Wainstock, Gali Pariente, Eyal Sheiner

**Affiliations:** 1 Department of Obstetrics and Gynecology, Faculty of Health Sciences, Soroka University Medical Center, Ben-Gurion University of the Negev, Beer-Sheva, Israel; 2 Department of Epidemiology and Health Services Evaluation, Ben-Gurion University of the Negev, Beer-Sheva, Israel

**Keywords:** Relaparotomy, Cesarean delivery, Risk factors, Pregnancy complications, Placental abnormalities, Cervical tear

## Abstract

**Purpose:**

Cesarean delivery (CD) is the most common obstetrical surgery with increasing rates worldwide. Although considered relatively safe, intra- and post-operative complications have been reported. One rare, but significant, complication after CD is relaparotomy. The present study was conducted to define risk factors for relaparotomy following a CD.

**Methods:**

A case–control study was conducted comparing all singleton CD that occurred in a tertiary medical center between the years 1991 and 2021. CDs complicated by relaparotomy (defined as the reopening of the fascia) were compared with CDs that were not complicated by relaparotomy. Generalized estimation equation (GEE) models were constructed to control for confounding variables.

**Results:**

During the study period, 49,922 CDs met our inclusion criteria, of them, 97 (0.2%) had undergone relaparotomy. The group of women complicated with relaparotomy tended to be multiparous and to have undergone a previous CD. Furthermore, these women had higher rates of placental complications (placenta previa, abruption and placenta accreta), preterm delivery, preeclampsia and chorioamnionitis. They also had higher rates of cervical tears and post-partum hemorrhage. Their neonates had lower birth weight and lower 5 min Apgar scores. In a GEE model, several independent risk factors for relaparotomy following CD were noted, with cervical tear being the most prominent (adjusted OR = 27.15, 95%CI 9.32 – 79.13, *p* < 0.001).

**Conclusion:**

Independent risk factors for relaparotomy following CD include cervical tear, placenta previa and accreta, placental abruption, preterm delivery, preeclampsia, and a previous CD. These risk factors should be taken into account when dealing with high-risk patients expected to undergo repeated CD.

**Supplementary Information:**

The online version contains supplementary material available at 10.1007/s00404-025-08199-w.

## Introduction

Cesarean delivery (CD) is the most common obstetrical surgery, with globally increasing rates. In 2015, 21.1% of the world’s births were CDs, almost double the number of CDs in 2000, and it increases annually by 4% [1]. While it is considered a relatively safe procedure, as with any surgery, CD is associated with short- and long-term risks that need to be taken into consideration, including infections, intra- and post-operative (post-partum) hemorrhage, bladder and intestinal injury, and cesarean hysterectomy [2–4]. Also, CD carries additional risk for subsequent pregnancies, such as abnormal placentation, uterine rupture, and all the added risks of having multiple CDs [5, 6]. Rates of severe maternal complications such as severe obstetric hemorrhage, pulmonary embolism, stroke, and other organ dysfunctions are two times greater for CD as compared with vaginal birth [3, 7, 8]. One rare, but substantial short-term complication of CD is the need for relaparotomy, occurring in 0.12–0.70% of CDs [9–11]. Relaparotomy is referred to as an abdominal operation performed after an initial surgery within 60 days, for complications arising following the primary surgery [12]. The main indication for relaparotomy is usually maternal instability due to post-partum complications, including post-partum hemorrhage or endometritis leading to maternal sepsis [9, 10].

Although relaparotomy is an important procedure needed in cases of potentially life-threatening complications of CD, it is considered a major complication itself due to its high mortality rates (as high as 9%) [9], intra- and post-operative complications, and further implications on both the patient and the medical team. Patients who undergo relaparotomy may present with additional complications such as bleeding, infections, coagulation disorders (including disseminated intravascular coagulation – DIC), need for blood transfusions, as well as an increase in hospitalization days and a burden to the medical system [13]. As such, identifying those at risk for such complications is crucial to optimize management and improve outcomes when facing such a scenario. Therefore, this study was designed to investigate and explore the risk factors associated with relaparotomy after CD.

## Methods

### Setting

This study was conducted at the Soroka University Medical Center (SUMC), a tertiary medical center. SUMC is the largest birth center in Israel that tends to the entire population residing in the Negev region, the southern part of Israel. As the only hospital in southern Israel, serving the entire obstetrical population in this area, this study represents nonselective population-based data.

The institutional review board (in accordance with the Helsinki declaration) approved the study that has been conducted according to the ethical standards established in the 1964 Declaration of Helsinki and in its later amendments (Helsinki Declaration 1975, revision 2013).

### Study population

The study population included all patients with a singleton pregnancy who underwent CD between the years 1991–2021 in SUMC. Fetuses with congenital anomalies and/or chromosomal abnormalities were excluded from the study.

### Study design

A case–control study was conducted comparing all women who had a singleton pregnancy and had delivery by CD. Women after CD complicated by relaparotomy were compared with women after CD without relaparotomy. Relaparotomy was defined as reopening of the fascia within 60 days after the initial surgery, for obstetrical-related complications following the primary surgery, excluding non-obstetrical indications for relaparotomy and laparoscopic re-entries.

Maternal data were collected from the SUMC OB/GYN database. This database holds maternal demographic and past obstetrical information, and detailed index pregnancy courses including pregnancy complications and perinatal, maternal, and fetal outcomes.

The following maternal characteristics were evaluated: maternal age, gravidity and parity, use of fertility treatments, obesity, smoking status, preexisting morbidity (including chronic hypertension and diabetes mellitus), and previous cesarean deliveries.

The following pregnancy complications were evaluated: hypertensive disorders, placenta previa, second trimester bleeding, placental abruption, chorioamnionitis, and preeclampsia with severe features.

The following perinatal outcomes were assessed: preterm delivery, low birth weight, low Apgar at 5 min, perinatal mortality, cervical tear, placenta accreta, post-partum hemorrhage, peri-partum hysterectomy.

### Statistical analysis

A univariate analysis was conducted comparing the maternal characteristics, pregnancy complications, and perinatal outcomes of the two groups, using the appropriate statistical tests: categorical variables were compared using the Chi-square test and are presented as frequencies and percentages. Continuous variables are presented using mean (± SD). Student’s *t* test was used in accordance with normal distribution.

A multivariable analysis was performed using a generalized estimating equations (GEE) was used to control for repeated deliveries by the same mother as well as for potential confounders based on clinical importance and the univariable analysis. All the models and statistical analysis were conducted with SPSS (version 29) software.

## Results

During the study period, 49,922 cesarean deliveries met our inclusion criteria. Of them, 97 (0.2%) had undergone relaparotomy. Maternal characteristics, pregnancy complications, and perinatal outcomes of women with and without relaparotomy after CD are listed in Table 1. The group of women requiring relaparotomy demonstrated distinct maternal and obstetric characteristics compared to those with uncomplicated CD. The group of women complicated with relaparotomy tended to be multiparous and to have undergone a previous CD. Furthermore, these women had higher rates of placental complications, including placental abruption, placenta previa, and placenta accreta. They also had higher rates of cervical tears and post-partum hemorrhage. Peri-partum hysterectomy was notably more frequent in the relaparotomy group. Adverse perinatal outcomes were also present at a higher rate among the relaparotomy cases: preterm delivery, low birth weight (< 2500 g), low Apgar scores at 5 min (< 7), and perinatal mortality cases were more common in the relaparotomy group (Table 1).

**Table 1.** Maternal characteristics, pregnancy complications, and perinatal outcomes of women with and without relaparotomy after cesarean delivery: a univariate analysis

	Relaparotomy *n* = 97	No relaparotomy *n* = 49,825	Odds ratio (95%CI)	*P* value
Maternal characteristics				
Maternal age, years (mean ± SD)	31.2 ± 5.8	30.4 ± 5.9		0.209
Parity*				
1	9 (9.3)	12,755 (25.6)		< 0.001
2–4	47 (48.5)	24,431 (49.0)		
5 +	41 (42.3)	12,634 (25.4)		
Previous cesarean delivery, *n* (%)	57 (58.8)	23,299 (46.8)	1.62 (1.08 – 2.43)	0.018
Pregnancy complications				
Hypertensive disorders^a^, *n* (%)	11 (11.3)	4868 (9.8)	1.18 (0.63 – 2.21)	0.603
Preeclampsia^b^, *n* (%)	10 (10.3)	1725 (3.5)	3.20 (1.66 – 6.17)	< 0.001
Diabetes mellitus^c^, *n* (%)	8 (8.2)	5166 (10.4)	0.77 (0.37 – 1.60)	0.494
Placenta previa, *n* (%)	17 (17.5)	1350 (2.7)	7.63 (4.51 – 12.91)	< 0.001
Second trimester bleeding, *n* (%)	1 (1.0)	26 (0.1)	19.95 (2.68 – 148.50)	< 0.001
Placental abruption, *n* (%)	13 (13.4)	1307 (2.6)	5.75 (3.19 – 10.33)	< 0.001
chorioamnionitis, *n* (%)	5 (5.2)	935 (1.9)	2.84 (1.15 – 70.00)	0.018
Perinatal outcomes				
Gestational age at birth, weeks (mean ± SD)	36.2 ± 4.0	38.1 ± 2.4		< 0.001
Preterm delivery, *n* (%)	41 (42.3)	7480 (15.0)	4.14 (2.76 – 6.20)	< 0.001
Birth weight, g (mean ± SD)	2756 ± 814	3112 ± 664		< 0.001
Low birth weight (< 2500 g.), *n* (%)	30 (30.9)	7122 (14.3)	2.68 (1.74 – 4.13)	< 0.001
Low Apgar at 5 min (< 7), *n* (%)	8 (8.7)	956 (1.9)	4.83 (2.33 – 10.00)	< 0.001
Perinatal mortality, *n* (%)	9 (9.3)	531 (1.1)	9.49 (4.75 – 18.95)	< 0.001
Cervical tear, *n* (%)	4 (4.1)	89 (0.2)	25.47 (9.15 – 70.87)	< 0.001
Placenta accreta, *n* (%)	11 (11.3)	196 (0.4)	32.38 (17.02 – 61.61)	< 0.001
Post-partum hemorrhage, *n* (%)	27 (27.8)	193 (0.4)	99.19 (62.24 – 158.06)	< 0.001
Peri-partum hysterectomy, *n* (%)	12 (12.4)	76 (0.2)	92.41 (48.49 – 176.12)	< 0.001

^a^Including chronic hypertension and gestational hypertension

^b^Including preeclampsia with and without severe features

^c^Including pre-gestational and gestational diabetes

^*^2 women from the study group and 5 women from the comparison group had missing data regarding parity

In a GEE model, several independent risk factors for relaparotomy following CD were noted (Table 2), with cervical tear being the most prominent one. The model adjusts for repeated deliveries of mothers, maternal age, chorioamnionitis, and second trimester bleeding. Other independent risk factors were placenta accreta, placental abruption, placenta previa, preterm delivery, preeclampsia, and previous CD (Table 2).

**Table 2.** Independent risk factors for relaparotomy after CD: Results from a GEE model

Characteristic	Adjusted* OR	95% confidence interval	*P* value
Cervical tear	27.15	9.32 – 79.13	< 0.001
Placenta accreta	10.23	3.86 – 27.15	< 0.001
Placental abruption	3.97	2.00 – 7.88	< 0.001
Placenta previa	2.95	1.30 – 6.68	0.009
Preterm delivery	2.34	1.39 – 3.94	0.001
Preeclampsia^a^	1.81	1.00 – 3.29	0.050
Previous CD	1.62	1.04 – 2.54	0.032

^*^The model adjusts for repeated deliveries, maternal age, chorioamnionitis and second trimester bleeding

^a^Including preeclampsia with and without severe features and eclampsia

## Discussion

Although rare, relaparotomy is a substantial complication of CD, with high mortality rates and complications of its own. As such, this study was designed to investigate possible risk factors for relaparotomy to help guide obstetricians and raise awareness for cases that may be more prone to encounter such a detrimental complication. We found that women who had a cervical tear during their CD had the highest risk for relaparotomy. Other risk factors included placental complications (including placental abruption, placenta previa, and placenta accreta), pregnancies complicated with preeclampsia or resulted in preterm delivery, and women who had a previous CD prior to the index pregnancy.

In our study, 0.2% of patients who delivered through CD had undergone relaparotomy. This incidence is in accordance with previous data, where the incidence rates ranged from 0.12 to 0.9% [9–11, 13–17]. The leading cause for relaparotomy in our study population was post-partum hemorrhage (PPH), responsible for 27.8% of all relaparotomies, with an OR of 99.19. It is not presented in the multivariable model, as this is a mediating variable and the cause for most relaparotomies. However, this finding is well supported by existing literature, being the leading cause of relaparotomy in many similar studies [9–11, 13–17], emphasizing the importance of meticulous management of blood loss in cesarean deliveries.

Cervical tears were the strongest independent risk factor for relaparotomy in our study. While this complication is well known in obstetric practices, mainly during vaginal deliveries, its direct association with relaparotomy has not been well studied. In our database, which comprises only women who delivered by CD, the diagnosis of a cervical tear during CD refers to an extension of the uterine incision involving the cervix. As an extremely vascular organ, an incisional extension to the uterine cervix poses an increased risk for post-partum hemorrhage (PPH) [18], which may ultimately result in a relaparotomy. This event differs from PPH caused by uterine atony and specifically targets a relatively uncommon surgical complication, highlighting this event as a risk factor related to the need for relaparotomy.

Placenta accreta, placental abruption, and placenta previa emerged as significant risk factors, reinforcing the well-established link between abnormal placentation and severe post-partum complications. Prior studies have consistently identified placental abruption and placenta previa as major risk factors for relaparotomy [9–11, 13–15, 17]. Weissmann-Brenner et al. found placenta previa to have the most prominent odds ratio (OR = 10.24) [13]. Seffah et al. specifically noted hemorrhage from the placental bed in cases of placenta previa [9], and Amikam et al. highlighted placental abruption as another major predictor (aOR = 4.62). While previous studies have focused primarily on placenta previa and placental abruption, our study adds to these findings by emphasizing placenta accreta as a particularly strong indicator (aOR = 10.23), which further highlights the link between placental abnormalities and the increased risk for women to undergo subsequent surgical interventions.

We identified previous CD as significant independent risk factor for relaparotomy, with an adjusted OR of 1.62. This finding is supported by previous literature, with an emphasis on multiple previous CDs [10, 14, 15, 17]. Given the increasing global rates of CD [1], this finding underscores the need for careful evaluation of its potential complications in recurrent CD.

Our findings demonstrate that hypertensive disorders of pregnancy (HDP), including preeclampsia, are significant risk factors for relaparotomy following CD. This observation is consistent with previous studies. Kessous et al. identified severe preeclampsia, among other conditions, as a significant contributor to the risk of relaparotomy [14]. Similarly, Burns et al. found hypertension to significantly increase the likelihood of relaparotomy (aOR = 1.83) [11]. In alignment with these findings, Amikam et al. reported HDP and placental abruption as independent risk factors, attributing the increased contribution partly to the higher incidence of PPH in these patients [17], while Denizli et al. also noted that most cases of relaparotomy were associated with severe preeclampsia or HELLP syndrome [16]. All in all, it is clear that hypertensive disorders complicating pregnancies also expose affected women to post-partum complications, including relatively rare post-surgical complications such as relaparotomy.

In our study, peri-partum hysterectomy was significantly more frequent in the group that underwent relaparotomy, observed in 12.4% of patients, compared to 0.2% in those who did not require a relaparotomy. The reported rates of hysterectomy among patients undergoing relaparotomy after cesarean delivery vary across studies. Kessous et al. reported a rate of 31.3%, Gedikbasi et al. observed 20%, and Seffah described a higher rate of 39%, while Denizli et al. and Amikam et al. reported lower rates of 4.3% and 10%, respectively [9, 10, 14, 16, 17]. Given that these, and our, studies were conducted in different countries and over a span of approximately 20 years, this variation likely reflects institutional differences, the evolution of clinical practice, and a shift to a more conservative approach in surgical interventions. Of particular relevance, the study by Kessous et al. was conducted in the same institution as ours over a decade ago, suggesting that changes in clinical protocols over time may have contributed to the lower hysterectomy rate in our cohort.

Our study has several important strengths. First, our large sample size and long study period over three decades provide a sufficient cohort for identifying risk factors, especially when considering the rarity of relaparotomy. In addition, the use of a comprehensive database that provided access to detailed records on pregnancy outcomes, as well as patients’ medical and obstetrical histories, allows a wider scope of pre- and post-delivery variables to be considered in the analysis.

We also acknowledge several limitations in our study as well. This research is retrospective and as such relies on existing data from medical records, which may be inconsistent or incomplete. However, the data was reported by an obstetrician immediately after the operation and surveillance and was routinely reviewed by skilled medical secretaries, making this potential bias less likely. Also, we conducted a meticulous analysis aiming to include as many potential confounders as possible and still able to find statistically significant results.

## Conclusions

Independent risk factors for relaparotomy following CD include cervical tear, placenta accreta, placental abruption, placenta previa, preterm delivery, preeclampsia, and a previous CD. These risk factors should be taken into account when dealing with high-risk patients, and specifically those expected to undergo repeated cesarean delivery. To be adequately prepared for possible subsequent relaparotomy, experienced obstetricians and gynecologists should be informed of these risk factors, in an effort to improve patient intra-operative and post-operative care.

## Supplementary Information

Below is the link to the electronic supplementary material.

Supplementary Material 1.

## Data Availability

We have full control of all primary data and agree to allow the journal to review the data if requested.
