# Supplementary material for: Exploring the risk factors for relaparotomy following cesarean delivery
Source: Arch Gynecol Obstet. 2025 Oct 3;312(6):2117–22. doi: 10.1007/s00404-025-08199-w (PMC12705735; doi:10.1007/s00404-025-08199-w)
Supplement: Supplementary file 1 — Supplementary Material 1. [file 404_2025_8199_MOESM1_ESM.docx]

**Table S1. Cesarean delivery indications in both study groups:**

| CD Indication | Relaparotomy (n=97) | No relaparotomy (n=49,825) | P Value |
| --- | --- | --- | --- |
| Labor dystocia (1^st^ and 2^nd^ stage), n (%) | 10 (10.3) | 7,339 (14.7) | 0.22 |
| Cord prolapse, n (%) | 4 (4.1) | 1,013 (2.0) | 0.14 |
| Pathological presentation, n (%) | 24 (24.7) | 12,332 (24.8) | 0.99 |
| Suspected placenta accreta, n (%) | 11 (11.3) | 196 (0.4) | <0.001 |
| Placental abruption, n (%) | 13 (13.4) | 1,307 (2.6) | <0.001 |
| Placenta previa, n (%) | 17 (17.5) | 1,350 (2.7) | <0.001 |
| Non-reassuring fetal heart rate, n (%) | 15 (15.5) | 9,083 (18.2) | 0.09 |
| Suspected fetal macrosomia, n (%) | 3 (3.1) | 3,811 (7.6) | 0.09 |
| Previous cesarean delivery, n (%) | 57 (58.8) | 23,299 (46.8) | 0.02 |
| Congenital uterine malformation, n (%) | 4 (4.1) | 1,114 (2.2) | 0.21 |
